# Evaluation of Protein Carbonyl Content in Healthy and Sick Hospitalized Horses

**DOI:** 10.3389/fvets.2020.582886

**Published:** 2020-10-27

**Authors:** Irene Nocera, Francesca Bonelli, Valentina Meucci, Riccardo Rinnovati, Alessandro Spadari, Luigi Intorre, Carlo Pretti, Micaela Sgorbini

**Affiliations:** ^1^Department of Veterinary Science, Veterinary Teaching Hospital, University of Pisa, Pisa, Italy; ^2^Department of Veterinary Medical Sciences, Ozzano Dell'Emilia, Bologna, Italy

**Keywords:** horse, systemic inflammatory response syndrome, protein carbonyl content, endotoxemia, biomarker

## Abstract

Literature on the protein carbonyl content (PCC) evaluation in horses is scarce, thus the aims were to evaluate the PCC in healthy and SIRS (Systemic Inflammatory Response Syndrome) horses and to investigate the performances of PCC in terms of sensitivity, specificity, and likelihood ratio in identifying SIRS positive and negative horses. A total of 72 adult horses were included. All the horses underwent to a complete physical examination, blood analysis, and were evaluated for the SIRS status. Blood samples were collected once in healthy horses and at admission time, then 24, 48, 72, and 96 h after admission in sick animals. PCC was evaluated using a method previously described. Data were statistically analyzed to verify differences in PCC between healthy vs. SIRS positive or SIRS negative horses at all sampling time. The receiver operating characteristic (ROC) curve was performed to verify sensitivity and specificity of PCC in the diagnosis of SIRS-positive and SIRS negative horses. The healthy horses were standardbred mares with a median age of 8.5 years. The sick horses were 31/54 females, 16/54 geldings, and 7/54 stallions of different breeds and with a median age of 12 years old. Eight out of 54 sick horses were SIRS negative, while 46/54 were SIRS positive. Statistically significant differences were obtained between healthy and SIRS positive horses, while no differences were observed between healthy and SIRS negative horses at any sampling time. The best cutoff value of PCC to discriminate between SIRS positive, SIRS negative, and healthy horses, the sensitivity and specificity of cutoff point, the area under receiver operating characteristic curve, the 95% confidence intervals, and the likelihood ratio were reported. We found higher PCC values in sick SIRS-positive horses vs. healthy ones with a decrement over time, while no differences at admission, nor during the observational period, were obtained in sick but SIRS-negative horses. The value of 0.049 nmol/ml/mg is reported as a potential cutoff for the diagnosis of SIRS positivity vs. healthy horses with a sensibility of 74.5% and a specificity of 72.2%. In conclusion, PCC seems to be a sensitive and specific marker for SIRS in horses.

## Introduction

Endotoxins [lipopolysaccharides (LPS)] are present in large quantities in the large bowel of horses but are harmless as long as they remain within the intestinal lumen. Equine gastrointestinal diseases impaired mucosal barrier and may lead to absorption of endotoxin and/or bacterial products through the mucosa into the bloodstream (1).

The term Systemic Inflammatory Response Syndrome (SIRS), rather than endotoxemia, is now used to describe the clinical status of endotoxemic horses. The physiologic changes associated with this inflammatory activation are alterations in the heart and respiratory rate, body temperature, mucous membrane status, and capillary refill time. Diseases that have been associated with SIRS in adult horses are especially those involving the gastrointestinal tract, such as the inflammatory intestinal diseases and strangulating obstructions (1). An early diagnosis should be the goal in the management of SIRS patients, allowing starting an adequate therapy in an early stage (2, 3).

During endotoxemia, neutrophils are induced by LPS to produce reactive oxygen species (ROS), playing a vital part in the systemic inflammatory process (4). Moreover, the oxidation of protein during inflammatory conditions, such as SIRS, is accompanied by the production of oxygen radicals that cause changes in the protein conformation. In particular, the change is characterized by the introduction of carbonyl groups (aldehyde and ketone) into the side chain of proteins by a variety of oxidation reactions (5). Carbonyl groups are present on proteins in normal tissues at low concentration (6). The measurement of the protein carbonyl content assesses the oxidative modification (7) in order to quantify the level of oxidative stress (OS) associated with inflammation (8).

Several studies have shown that the action of ROS on proteins results in the formation of carbonyl groups [protein carbonyl content (PCC)] (8). The protein carbonylation is generated through various mechanisms, which include neutrophil-derived hypochlorous acid and conjugation to aldehyde products of lipids (9). The carbonylated proteins have a long half-life; therefore, evaluation of the carbonyl group content provides a significant indication to the amount of oxidative stress under disease conditions (8).

Literature on the PCC evaluation in horses is scarce. The study by Dimock et al. (10) evaluated the PCC in the synovial fluid of healthy and diseased horses' joints. The authors found higher PCC in the pathologic joints respect to the healthy ones.

The aims of the present work were to assess the PCC in healthy and SIRS horses in order to evaluate the differences between the groups and to investigate the performances of PCC in terms of sensitivity, specificity, and likelihood ratio in identifying SIRS positive and negative horses.

## Materials and Methods

The present *in-vivo* prospective study was approved by the Institutional Animal Care and Use Committee of the University of Pisa (Prot. N. 23506/15), and an owner's written consent was obtained for the collection of blood for all the horses included in this study. The study took place during 2017–2018.

A total of 72 adult horses were included. Eighteen out of 72 (22%) were healthy mares kept at the Breeding Stud Farm of the Department of Veterinary Sciences of the University of Pisa as embryo transfer recipients, while 54/72 (75%) were sick horses referred to two different veterinary teaching hospitals (VTHs) providing secondary health care. Sick horses were enrolled at admission time at the VTHs.

All the horses underwent to a complete physical examination and blood work analysis, plus collateral examination if needed. Moreover, the following data were recorded in order to evaluate the SIRS status (11): presence of abnormal leukocyte count or distribution as leukopenia, leukocytosis or >10% band neutrophils (lower than 5 or higher than 12.5 × 10^3^ μL), hyperthermia or hypothermia (lower than 37 or higher than 38.5°C), tachycardia (>52 bpm), and tachypnea (>20 bpm). Those animals presented a normal physical examination, laboratory data within reference ranges, and a SIRS score = 0 were included in the control group. This latter group was free from internal and external parasites and was not treated pharmacological at least 15 days prior to the study.

Horses were considered sick on the basis of clinical signs, blood work, diagnostic imaging, and collateral exams. Also, sick horses with 0 or 1 abnormal criterion were considered SIRS-negative, while sick horses with 2 or more abnormal criteria were included in the SIRS positive group (11).

Along with data recorded for SIRS evaluation, blood samples were collected for complete blood count, and PCC analysis from the jugular vein of each animal included using a sterile syringe and 16G needle. Samples were withdrawn once in healthy horses, contextually to the physical examination and blood work analysis, while in sick horses, blood was collected at admission time (T0), then 24 (T1), 48 (T2), 72 (T3), 96 (T4) h after admission. After the first sampling (T0), all the horses received appropriate treatments based on the clinical signs and the diagnosis. Each blood sample was divided in two aliquots: 1 ml in aliquot was collected in a potassium ethylene diamine tetra acid (K_2_EDTA) test tube and analyzed by a cell counter (ProCyte Dx™, IDEXX, USA) within 5 min after the collection. A second 2.5 ml aliquot was collected in LH-heparin tubes and centrifuged at 3,500 relative centrifugal force for 10 min within 30 min of collection. The harvested plasma was placed in sterile tubes, frozen at −80°C. PCC was measured in a single batch as described below.

It was not possible to standardize the time elapsed from the onset of clinical signs and the time of admission and first sampling. Moreover, for the sick horses, it was not possible to collect a complete history regarding the preadmission treatments or the presence of internal or external parasites. Moreover, some sick horses have been discharged or euthanized before T4, so it was not possible to collect samples through the whole study period (T0–T4) for all the patients included.

### PCC Evaluation

PCC was evaluated on a total of 192 plasma samples using a previously described method (12) based on the spectrophotometric detection of the reaction between 2.4-dinitrophenyl hydrazine with protein carbonyls to form protein hydrazone. The results were expressed as nmol/ml/mg protein of PCC by using wavelength 370 nm. The protein concentrations were determined by the Lowry (13) method using bovine serum albumin as a standard.

### Statistical Analysis

Data were analyzed for distribution using the D'Agostino-Pearson test and results were expressed as median, standard error, minimum, and maximum values.

Kruskal-Wallis and Dunn's multiple comparisons tests were used to verify differences in PCC values between healthy vs. SIRS positive or SIRS negative horses at all sampling time.

The receiver operating characteristic (ROC) curve was performed to verify sensitivity and specificity of PCC in the diagnosis of SIRS-positive and SIRS negative horses. Statistical analysis was performed using a commercial software (GraphPad Prism 6.0, USA) and the statistical significance was set at *p* < 0.05.

## Results

### Caseload

The healthy horses were standardbred mares with a median age of 8.5 years old (3–19 years old). The sick horses were 31/54 (57%) females, 16/54 (30%) geldings, and 7/54 (13%) stallions. Breeds were Standardbred (*n* = 4), cross breed (*n* = 6), Quarter Horse (*n* = 7), Thoroughbred (*n* = 1), Pony (*n* = 8), Arabian (*n* = 8), Warmblood (*n* = 15), Draft (*n* = 5). The median age was 12 years (1–32 years old).

The 54 sick horses were grouped as follow on the basis of the final diagnosis: obstructive non-strangulative colic (*n* = 26); obstructive strangulative colic (*n* = 17); other gastrointestinal diseases [*n* = 3: 1/3 abdominal effusion, Equine Gastric Ulcer Syndrome (EGUS) and hepatic failure, respectively]; reproductive diseases (*n* = 4: 2/4 dystocia, 1/4 uterine torsion and hematoma, respectively); musculoskeletal and/or skin trauma/disease (myopathy) (*n* = 4).

Eight out of 54 (14.8%) sick horses were SIRS negative, while 46/54 (85.2%) were SIRS positive. The SIRS negative horses were affected by obstructive non-strangulative (7/8, 87.5%), and reproductive diseases (uterine torsion) (1/8, 12.5%).

The SIRS positive horses were affected by obstructive non-strangulative (21/46, 45.6%) and strangulative (15/46, 32.6%) colic, musculoskeletal and/or skin trauma/disease (myopathy) (4/46, 8.7%), other gastrointestinal diseases and reproductive diseases, respectively (3/46, 6.5%, respectively).

None of the sick horses spontaneously died, while 39 out of 54 (72.2%) were discharged and 15/54 horses (27.8%) were humanely euthanized due not for economic reasons.

Of the 39 horses discharged, 7/39 (17.9%) were SIRS negative and 32/39 (82.6%) were SIRS positive; 24/39 (61.6%) were affected by obstructive non-strangulative and 7/39 (17.9%) by obstructive strangulative colic, 3/39 (7.7%) by reproductive diseases, trauma and/or musculoskeletal disease (myopathy), respectively, and 2/39 (5.1%) by other gastrointestinal diseases.

Of the 15 horses euthanized, 1/15 (6.7%) was SIRS negative, while 14/15 (93.3%) were SIRS positive subjects; 10/15 (66.7%) were affected by obstructive strangulative colic, 2/15 (13.3%) by other gastrointestinal diseases and reproductive diseases, respectively, and 1/15 by and obstructive non-strangulative (6.7%). Twelve out of 15 (80%) horses were euthanized after the first sampling, 1/15 (6.7%) after the second and 2/15 (13.3%) after the last sample.

### PCC Results

**Figure 1:**
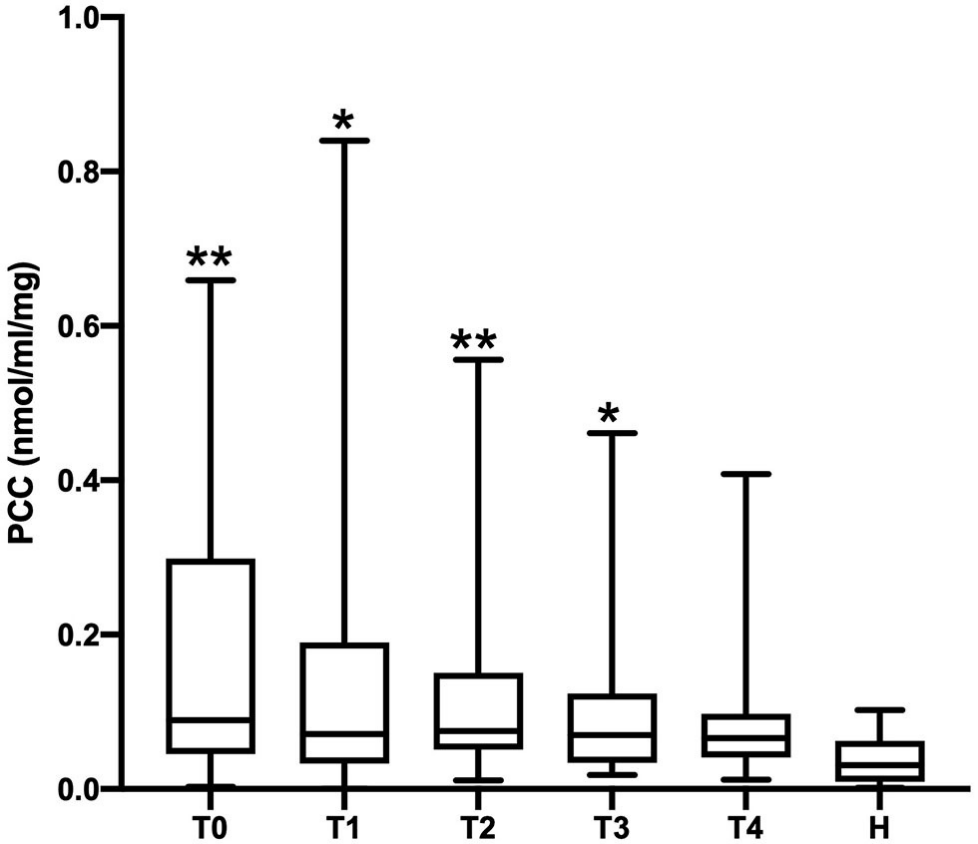
Dot plots displaying the PCC values in healthy and SIRS-positive horses. The different upper-case symbols above the dot plots denote a significant difference: ^*^P < 0.05 respect to H; ^**^P < 0.01 respect to H). The horizontal lines represent the median, minimum, and maximum values. H: healthy horse groups; T0, T1, T2, T3, T4: SIRS positive horse groups sampled at admission in VTHs (T0), 24 (T1), 48 (T2), 72 (T3), 96 (T4) h after admission.

**Figure 2:**
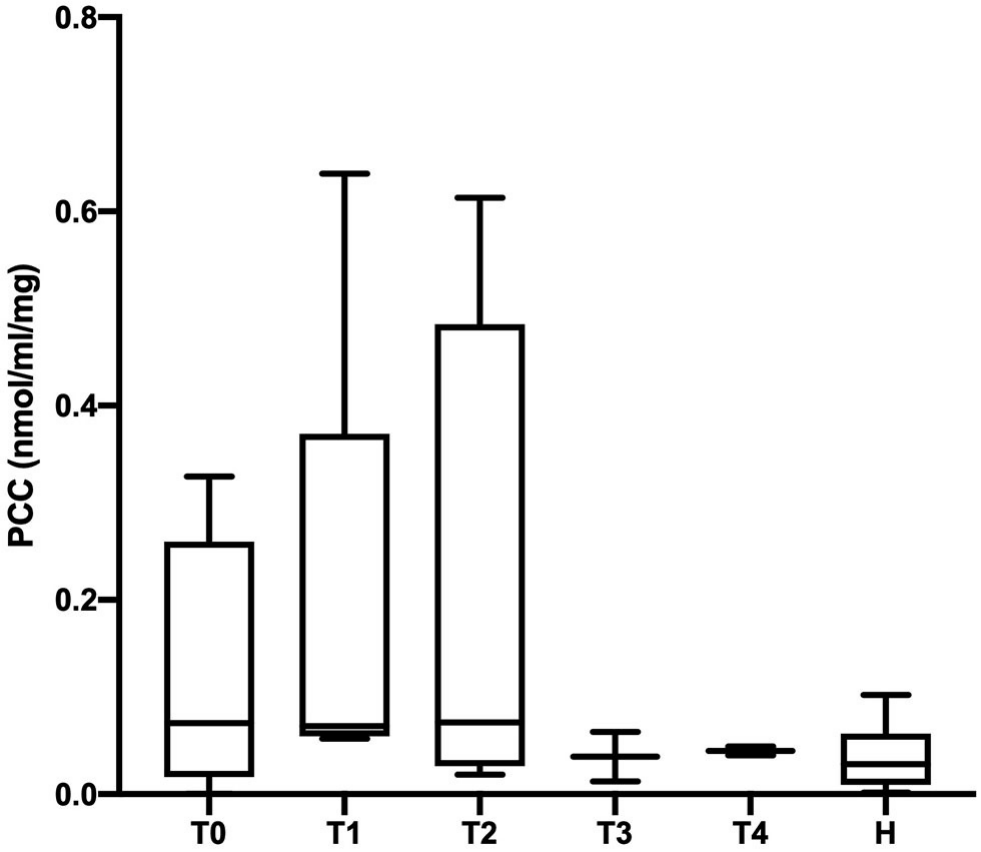
Dot plots displaying the PCC values in healthy and SIRS-negative horses. The horizontal lines represent the median, minimum, and maximum values. H: healthy horse groups; T0, T1, T2, T3, T4: SIRS-negative horse groups sampled at admission in VTHs (T0), 24 (T1), 48 (T2), 72 (T3), 96 (T4) h after admission.

The PCC (nmol/mL/mg protein) values obtained in healthy, SIRS negative, and SIRS positive horses are reported in Table 1. Statistically significant differences were obtained between healthy and SIRS positive horses at T0, T1, T2, and T3 (Figure 1), while no differences were observed between healthy and SIRS negative horses at any sampling time (Figure 2).

**Table 1:** PCC (nmol/mL/mg protein) values obtained in healthy and SIRS-negative or SIRS-positive horses at admission in VTHs (T0), 24 (T1), 48 (T2), 72 (T3), and 96 (T4) h after admission.

	**T0**			**T24**		**T48**		**T72**		**T96**	
	**Healthy**	**SIRS**	**SIRS**	**SIRS**	**SIRS**	**SIRS**	**SIRS**	**SIRS**	**SIRS**	**SIRS**	**SIRS**
	**(*n* = 18)**	**negative**	**positive**	**negative**	**positive**	**negative**	**positive**	**negative**	**positive**	**negative**	**positive**
		**(*n* = 8)**	**(*n* = 46)**	**(*n* = 5)**	**(*n* = 31)**	**(*n* = 4)**	**(*n* = 29)**	**(*n* = 2)**	**(*n* = 26)**	**(*n* = 2)**	**(*n* = 21)**
Me	0.031	0.073	0.089	0.070	0.071	0.074	0.075	0.038	0.070	0.044	0.070
m	0.001	0.000	0.003	0.057	0.000	0.020	0.011	0.013	0.018	0.040	0.012
M	0.102	0.327	0.658	0.639	0.840	0.614	0.556	0.064	0.461	0.049	0.408
CI 95%	0.010–0.058	0.000–0.327	0.059–0.216	0.057–0.638	0.035–0.147	0.020–0.613	0.057–0.132	00.012–0.064	0.036–0.106	0.039–0.049	0.043–0.096

*Me, Median value; m, minimum value; M, maximum value; CI median confidence interval at 95%*.

The best cutoff value of PCC to discriminate between SIRS positive, SIRS negative, and healthy horses, the sensitivity and specificity of cutoff point, the area under ROC curve, the 95% confidence intervals, and the likelihood ratio are reported in Table 2.

**Table 2:** Sensitivity and specificity of cutoff point of the PCC to discriminate between SIRS and healthy horses.

	**AUC**	**95% CI**	***p***	**Cut-Off**	**Se (%)**	**Sp (%)**	**LR**
SIRS positive/healthy	0.77	0.65–0.88	<0.001	>0.049	74.47 (60.49–84.75)	72.22 (49.13–87.50)	2.681
SIRS negative/healthy	0.63	0.33–0.94	>0.05	>0.059	71.43 (35.89–94.92)	77.78 (54.79–91.00)	3.214

*AUC, area under receiver operating characteristic curve; CI, confidence interval; Se, sensitivity; Sp, specificity; LR, Likelihood ratio*.

## Discussion

The aims of the present work were to evaluate the PCC in healthy and SIRS horses in order to evaluate the differences between the groups and to investigate the performances of PCC in terms of sensitivity, specificity, and likelihood ratio in identifying SIRS positive and negative horses.

Overall, we found higher values of PCC in sick SIRS-positive horses respect to healthy ones with a decrement in PCC values over time. On the other hand, no differences at admission, nor during the observational period were obtained in sick but SIRS-negative horses. Moreover, cutoff values to diagnose SIRS-positive horses were reported. To the authors' knowledge, this is the first report on the evaluation of PCC in horses affected by systemic diseases.

In the present study, an increase in protein oxidation was found in SIRS-positive horses with respect to SIRS-negative or healthy subjects at admission to the VTHs (T0). Our results are in line with a previous study performed in dogs (14). In both the studies, no differences were reported between the two groups of sick animals (septic and non-septic dogs, SIRS, and no-SIRS horses), while differences were found for dogs with sepsis or inflammation or for horses with inflammation (SIRS positive) respect to the healthy ones. Our results did not show differences between sick SIRS-negative horses and controls. Lack of difference between the healthy horses and the sick SIRS-negative ones could be due to the correlation between the increasing of PCC values and the severity of the inflammatory status and clinical outcome, as already reported in men (7). Our findings are also in line with studies performed in human medicine in which the authors compared septic patients or patients affected by major trauma and reported statistical differences in the PCC values between the sick patients and the healthy ones at the time of admission to the hospital (7, 15, 16).

In this study, we found differences between healthy and sick SIRS-positive horses at admission to the VTHs and then at 24- and 48-h post-admission, while no differences were found at the other timepoints (72- and 96-h post-admission). This support the hypothesis that medical treatment and/or colic surgery solved the primary problem and the oxidation of plasma proteins decreased over time reaching values similar to healthy horses. In human medicine, previous studies investigated the trend of the PCC over a 10-day period in patients affected by sepsis or major trauma, reporting a decrement over time. The PCC values were significantly higher in sick patients compared to the control group throughout the whole study period (7, 15). This discrepancy could be due to the different patients involved (men vs. horses) but also to the different inclusion criteria used. In particular, the studies on humans included severe septic patients or patients with major trauma, with an Injury Severity Score (ISS) ranging between 26 and 50. In humans, the ISS or the New Injury Severity Score (NISS), is correlated with the SIRS severity, which may have influenced the decrement of the PCC during the time (17). In our study, we did not evaluate the SIRS score and the severity of SIRS but only the SIRS status (SIRS positive or negative). Thus, it might be possible that our population included less severely sick patients compared to human studies.

The ROC curve analysis revealed a PCC value of 0.049 nmol/ml/mg as a potential cutoff for the diagnosis of SIRS positivity vs. healthy horses with a sensitivity of 74.5% and a specificity of 72.2%. A limitation of this study is the sample size, especially in the healthy group, that might have influenced the not high values in sensitivity, specificity, and likelihood ratio. Ruggerone et al. (14) reported that PON-1 may discriminate dogs with sepsis with non-septic dogs affected by inflammation or healthy dogs. Our results seem to be in line with the previous study (14), but the authors did not report a cutoff value or sensitivity and specificity assessment.

In humans, higher values of PCC have been associated with mortality in septic patients (16). In this study, the evaluation of the PCC levels and the mortality was not assessed.

In conclusion, PCC seems to be a sensitive and specific marker for SIRS in horses. The limit of the study might be the number of cases included (*n* = 54 sick horses). An increased study population could be needed to verify the sensitivity and specificity of the cutoff obtained, to assess if the specific oxidative marker reflects the severity of the disease (i.e., assessing the SIRS score and the marker level) and to verify the prognostic relevance in terms of mortality/survival.

## Data Availability Statement

The raw data supporting the conclusions of this article will be made available by the authors, without undue reservation.

## Ethics Statement

The present *in vivo* prospective study was approved by the Institutional Animal Care and Use Committee of the University of Pisa (Prot. N. 23506/15) and an owner's written consent was obtained for the collection of plasma for all the horses included in this study.

## Author Contributions

IN, FB, VM, and MS contributed to the conception and design the study, wrote the first draft of the manuscript, and provided a critical revision. RR and AS provided critical revision. LI and CP revised the section related to the PCC evaluation. All authors contributed to the article and approved the submitted version.

## Conflict of Interest

The authors declare that the research was conducted in the absence of any commercial or financial relationships that could be construed as a potential conflict of interest.

## Abbreviations

LPS: lipopolysaccharides
SIRS: systemic inflammatory response syndrome
ROS: reactive oxygen species
OS: oxidative stress
PCC: protein carbonyl concentration
VTH: veterinary teaching hospital
K_2_EDTA: potassium ethylene diamine tetra acetic.
